# High Endothelial Venules: A Vascular Perspective on Tertiary Lymphoid Structures in Cancer

**DOI:** 10.3389/fimmu.2021.736670

**Published:** 2021-08-17

**Authors:** Gerlanda Vella, Sophie Guelfi, Gabriele Bergers

**Affiliations:** ^1^Laboratory of Tumor Microenvironment and Therapeutic Resistance, Department of Oncology, Vlaams Instituut voor Biotechnologie (VIB)-Center for Cancer Biology, Katholieke Universiteit (KU) Leuven, Leuven, Belgium; ^2^Department of Neurological Surgery, UCSF Comprehensive Cancer Center, University of California San Francisco (UCSF), San Francisco, CA, United States

**Keywords:** high endothelial venules, tertiary lymphoid structures, tumor endothelial cells, tumor immunity, immunotherapy, lymphotoxin beta receptor, sentinel lymph node, metastasis

## Abstract

High endothelial venules (HEVs) are specialized postcapillary venules composed of cuboidal blood endothelial cells that express high levels of sulfated sialomucins to bind L-Selectin/CD62L on lymphocytes, thereby facilitating their transmigration from the blood into the lymph nodes (LN) and other secondary lymphoid organs (SLO). HEVs have also been identified in human and murine tumors in predominantly CD3^+^T cell-enriched areas with fewer CD20^+^B-cell aggregates that are reminiscent of tertiary lymphoid-like structures (TLS). While HEV/TLS areas in human tumors are predominantly associated with increased survival, tumoral HEVs (TU-HEV) in mice have shown to foster lymphocyte-enriched immune centers and boost an immune response combined with different immunotherapies. Here, we discuss the current insight into TU-HEV formation, function, and regulation in tumors and elaborate on the functional implication, opportunities, and challenges of TU-HEV formation for cancer immunotherapy.

## Introduction

### Tumoral Angiogenesis and Immune Escape

**Figure 1:**
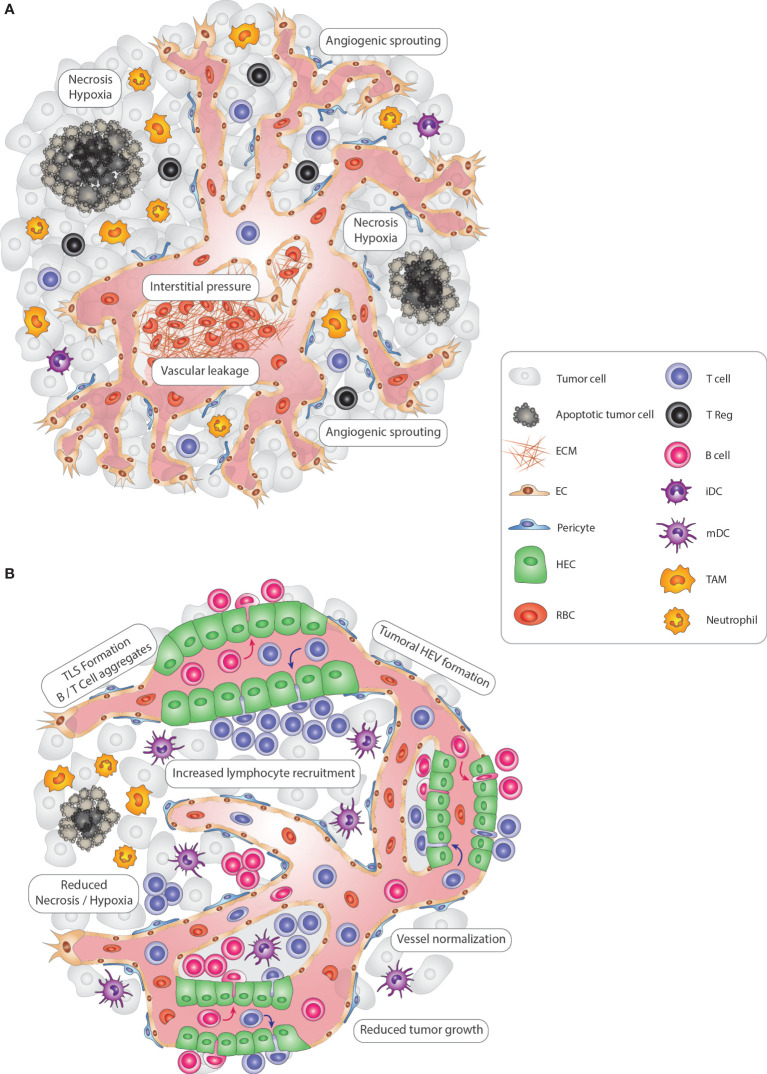
Modulating vascular-immune interactions in solid tumors *via* TLS and HEV formation. **(A)** In solid tumors, vascular-immune interactions promote immunosuppression and neovascularization to allow the growth of cancer cells. Continuous angiogenic sprouting of ECs leads to an abnormal, less mature tumor vasculature with poor pericyte coverage leading to leakiness, dysfunctional blood flow and increased interstitial pressure which in turn promotes hypoxia and necrosis. Importantly, tumor blood vessels convey immunosuppressive signals that inhibit CD4^+^ and CD8^+^ lymphocyte infiltration, DC maturation and activate immunosuppressive regulatory T-cells (Tregs). Finally, innate immune cells, including TAMs and neutrophils, also suppress immunosurveillance and promote vascular remodeling. **(B)** Tumoral TLS and HEV induction promote anti-tumor immunity. In an immune-stimulatory setting, the tumor vasculature becomes transiently normalized with increased pericyte coverage, thus re-establishing blood flow and perfusion and reducing hypoxic and necrotic areas of the tumor. Due to the enhanced functionality, vessels are angiostatic and more prone to recruit immune cells which can lead to the formation of HEVs. Subsequently, HEV-containing TLSs form, with immune cell centers composed of CD4+ and CD8+ T, B lymphocytes, and mature DCs that promote an anti-tumor immune response. Tregs, TAMs, and neutrophils are less abundant, thus no longer exerting an immunosuppressive function. Altogether, re-awakening and boosting the immune system *via* TLS and HEV formation leads to reduced tumor cell growth and is ultimately beneficial for cancer progression. ECM, extra-cellular matrix; EC, endothelial cell; TLS, tertiary-lymphoid structure; HEV, high endothelial venule; HEC, high endothelial cell; iDC, immature dendritic cell; mDC, mature dendritic cell NK, Natural Killer cell; TAM, tumor-associated macrophage; RBC, red blood cell.

Solid tumors are heterogeneous and complex cellular ecosystems in which cancer cells shape their microenvironment to their advantage by actively remodeling the local immune, vascular and stromal compartments (1). Thus, tumors have also been considered as “wounds that never heal” because they increasingly promote immunosuppression and neovascularization to sustain the rapid growth of cancer cells (2, 3). Due to the anomalous proangiogenic signals, these tumors exhibit a continuously growing tumor vasculature with a chaotic composition of venules, postcapillary venules, arterioles, and capillaries. Consequently, angiogenic tumor vessels typically exhibit abnormal structural and functional characteristics of poor vessel maturation, leakiness, and staggered blood flow due to the elevated interstitial pressure (4–6) (**Figure 1**). With these vascular aberrations, hypoxic, acidic, and necrotic regions appear in tumors that induce an additional wave of proangiogenic signals, exacerbating disease because they support metastasis by enabling tumor cell intravasation into the bloodstream and obstructing adequate delivery of anti-cancer drugs (4, 7). Importantly, as part of the wound repair program, angiogenic factors including vascular endothelial growth factor (VEGF) and angiopoietins also convey immunosuppressive signals. They reduce the expression of ICAM1 and VCAM1 lymphocyte adhesion molecules in endothelial cells that limit vascular adhesion of lymphocytes and subsequent infiltration into the tumor (8, 9). Further, VEGF can directly inhibit dendritic cell (DC) maturation and activate antigen-specific regulatory T-cells (8, 9). Tumor-recruited innate immune cells, including macrophages, myeloid-derived suppressor cells (MDSC), and neutrophils, are an additional source of angiogenic and immunosuppressive factors to suppress immunosurveillance and promote vascular and matrix remodeling **(** **Figure 1** **)** (3, 10). Thus, tumors employ multiple mechanisms of the tissue repair program to keep their environment in a favorable, immunosuppressive and angiogenic state.

### TLSs in Tumors

The *in situ* detection of tumor-infiltrating lymphocytes has been commonly used in the clinic because the degree of CD8 T cell infiltration often correlates with patient survival (11). Such histopathological studies revealed substantial lymphocyte aggregates in some tumors of patients who had a predominantly favorable outcome compared to those who did not. These structures display variably organized T- and B cell aggregates, sometimes even a T cell-rich zone with mature DCs juxtaposing a B cell follicle with germinal center characteristics. They are commonly located at the tumor interphase or in adjacent areas to the tumor and entail blood and lymphatic vessels and other stromal cells that are commonly observed in secondary lymphoid organs (SLOs). Indeed, due to their resemblance with SLOs, these ectopic lymphoid-like structures have been coined tertiary lymphoid structures (TLS) and have been observed in the pathological contexts of chronic inflammatory and autoimmune diseases (12, 13); including rheumatoid arthritis (14, 15), autoimmune thyroiditis (16), inflammatory bowel disease (17, 18), and *H. pylori gastritis* infections (19, 20). The reader can refer to (21–23) for their detailed description. Under these conditions, TLSs are abnormal structures of an active immune response against self-antigen, promote autoimmune reactions, and subsequently aggravate the disease. Since TLSs in solid tumors are mostly associated with improved tumor response, it is conceivable that they are also sites of activated lymphocytes generating an immune response (22). This raises the question as to how lymphocytes can preferentially infiltrate these locations despite the presence of an overall immunosuppressive vascular environment.

### TLSs, Like SLOs, Contain High Endothelial Venules

While histopathological studies have extensively characterized immune infiltrates and defined tumoral TLSs in human cancer for the last 30 years (22), less is known regarding the vascular components of tumoral TLSs. TLS vessels present a resemblance to those in lymph nodes and other SLOs. Lymphatic vessels (LV) have been identified around multiple TLSs and are recognized by the typical lymphatic markers such as LYVE-1, PROX-1, and podoplanin (24). LVs remove interstitial fluid (containing plasma proteins, lipids etc.) that extravasate from blood capillary filtrates back into the blood circulation. They serve as the main route for dendritic cells, antigens, and inflammatory mediators into the lymph node (LN) and are essential players in peripheral tolerance, immunosurveillance, and resolution of inflammation (25). Only about a decade ago, Martinet and colleagues made the first observations of unusual blood vessels in human solid cancer samples which resembled high endothelial venules (HEV) in SLOs (26). HEVs are morphologically and functionally specialized blood vessels that deliver naïve lymphocytes from the bloodstream into the LN, in which lymphocytes become primed and educated by antigen-presenting cells (APC) (e.g., DCs) (**Figure 1**). Lymphocytes exit then through efferent LVs, which lead into the blood vascular system *via* the thoracic duct to circulate the cells through the body (27–31).

These observations beg the question as to whether HEVs and LVs in TLSs play comparable roles and are regulated similarly to those in LNs. In this review, we will focus on recent advances in HEV formation, function, and regulation in the tumoral context. From observations in human cancer, we will highlight studies of intratumoral HEVs in several mouse cancer models and describe the morphological and functional HEV alterations in premetastatic and metastatic LNs. Finally, we will discuss the functional implication, opportunities, and challenges of tumoral HEV formation for cancer immunotherapy.

## Physiological HEVs in SLOs

### Characteristics of HEVs

HEVs develop from postcapillary venules in LN, Peyer’s patches (PPs), and other SLOs but are absent in the spleen. In SLOs, adaptive immune responses are initiated through the active recruitment of naïve lymphocytes, which is facilitated by HEVs. In contrast to the common flat appearance of endothelial cells (EC), ECs lining HEVs have a cuboidal appearance with a prominent glycocalyx for which they have been coined “high” endothelial cells (HEC) (27, 32). HEVs form a thick basal lamina and are encompassed by a perivascular sheath of fibroblastic reticular cells (FRC) (33–36). Due to their specialized function as lymphocyte portals, HEVs express high levels of the ligand of L-Selectin/CD62L, the classic homing receptor for T and B-lymphocytes. L-Selectin ligands are sialomucins that entail sulfated mucin-like glycoproteins, including podocalyxin, endomucin, CD34, nepmucin, and GlyCAM-1 (rodent-specific) (30, 37). Importantly, sialomucins bind more effectively L-selectin after HEV-specific post-translational modifications by sulfotransferases and glycosyltransferases, including Carbohydrate Sulfotransferase 4 (CHST4) (38, 39) and Alpha-(1,3)-Fucosyltransferase VII (FucT-VII) (40, 41), which are highly expressed in HECs but not in other endothelial cells. Thus, antibodies have been developed that recognize HEVs by binding to these modified sialomucins. The most prominent antibody MECA79 (“Mouse Endothelial Cell Antigen-79”) detects sulfated peripheral node addressin (PNAd) that is decorated with the carbohydrate element 6-sulfo sialyl Lewis^X^, (sialic acidα2-3Galβ1-4(Fucα1-3(sulfo-6) GlcNAcβ1-R) (37, 42, 43). Aside from these HEV-specific characteristics, HEVs express different vascular addressins in an SLO-dependent manner. While PNAd^+^ HEVs are mainly found in peripheral LNs (pLN), HEVs in PPs express mucosal addressin cell adhesion molecule-1 (MAdCAM-1) but not PNAd (44). Notwithstanding, neonatal pLN-HEVs first express MAdCAM-1 for 3-4 weeks after birth before they switch to the PNAd^+^ phenotype concomitant with HEV maturation, suggesting that MAdCAM-1 is an immature HEV marker in pLNs (45). Finally, mesenteric, sacral, and cervical LN-HEVs display expression of both PNAd and MAdCAM-1 vascular addressins (46, 47). It is noteworthy that most of our knowledge of HEV biology is derived from studies on pLNs.

### HEVs Facilitate the Transmigration of Lymphocytes

The detailed migration process of lymphocytes across endothelial cells, including HEVs has been thoroughly studied by intravital microscopy (48, 49). This multistep event of lymphocyte tethering, rolling, sticking, and transmigration is tightly regulated by a coordinated interplay of adhesion molecules, integrins, and chemokines (37, 45, 48, 50). Migration of naïve and central memory T cells, as well as naïve B cells, starts with the binding of L-selectin to the 6-sulfo sialyl Lewis^X^ on the HEV walls. This tethering interaction reduces lymphocyte rolling and enables binding to the chemokines CCL19, CCL21, CXCL12, and CXCL13, which are presented on the luminal surface of HEVs, *via* CCR7, CXCR4, and CXCR5 receptors (51–53).

The chemoattractant-chemoattractant receptor axes that predominately govern the trafficking of lymphocytes into and out of LNs are CCL19/CCR7 and sphingosine 1-phosphate (S1P)/sphingosine 1 receptor 1 (S1PR1), respectively (30, 54). Blood-borne lymphocytes downregulate S1PR1 and use CCR7 signaling to adhere to HEVs for transmigration. During their LN residency, recirculating lymphocytes reacquire S1PR1 and attenuate their sensitivity to chemokines. Eventually, lymphocytes exit the LN by entering the cortical or medullary lymphatics, a process that depends upon S1PR1 signaling. Upon entering into the lymph, lymphocytes lose their polarity, downregulate their sensitivity to S1P due to the high concentration of S1P, and upregulate their sensitivity to chemokines (55). However, many of the details of lymphocyte transmigration across endothelial barriers remain poorly understood.

The integrin lymphocyte function-associated antigen 1(LFA-1/αLβ2) on lymphocytes interacts with the ICAM1 and ICAM2 adhesion molecules on the HEV surface, which leads to a firm arrest and subsequent paracellular or transcellular lymphocyte transmigration into the LN parenchyma (56, 57). Another notable characteristic of HEVs is their ability to form HEV pockets in which lymphocytes can be temporarily retained before their egress (56, 58). Although their function remains obscure, it is tempting to speculate that they exhibit specific lymphocyte communication centers and/or form when an overflow of lymphocytes arrives.

While HEVs typically recruit naïve and central memory lymphocytes in homeostatic LNs (30), HEVs of inflamed LNs become phenotypically remodeled, expand, and are capable of recruiting novel immune subsets into the LN. Specifically, they upregulate P-selectin, E-selectin, and CXCL9 and appear less mature because they induce gene expression of MAdCAM-1 while reducing *Fut7* and *Glycam1* transcription (59–61). Inflamed LN-HEVs can therefore recruit activated/effector T cells, plasmacytoid DCs, monocytes and neutrophils, whereas their capability to enroll naïve lymphocytes is not compromised (59, 60, 62–64)

### HEV Regulation and Signaling in Lymph Nodes

The development of LNs is a well-organized event that involves the crosstalk between the hematopoietic lymphoid tissue inducer (LTi) cells and the mesenchymal lymphoid-tissue organizer (LTo) cells (65). It is thought that HEVs develop concomitantly with the accumulation of LTi cells to form the lymphoid anlagen; however, the developmental ontogeny of HEVs in lymphoid organs as well as the stepwise transcriptional program of HEV specification has not been clearly identified so far (66). The most important signaling pathway that has been directly linked to developmental LN-HEV formation and maintenance is the lymphotoxin- (LT)/lymphotoxin β receptor (LTβR)–signaling pathway (67–69). LTβR is a member of the TNF receptor superfamily which binds the LTα1β2 heterotrimers or LIGHT (“homologous to **l**ymphotoxin, exhibits **i**nducible expression and competes with HSV **g**lycoprotein D for binding to **h**erpesvirus entry mediator, a receptor expressed on **T** lymphocytes”) also known as TNSF14 (tumor necrosis factor superfamily member 14). Although the LTβR can activate both the canonical and non-canonical NFκB pathways, the non-canonical axis appears to be preferentially activated, specifically through the NIK kinase and the RelB/p52 transcriptional complex (70). Deletion of LTβR in ECs impaired the formation of HEVs in LN and subsequently LN homeostasis (69).

More recently, the S1P/S1PR1 axis has also been proposed to regulate HEV integrity in an autocrine manner and to facilitate HEV-DC interactions in LNs (71), thus suggesting the involvement of alternative signaling pathways regulating LN-HEV maintenance.

## High Endothelial Venules in Human Cancer

Martinet and colleagues made the first and formal observations of ectopic HEVs in human cancer samples (26). They observed MECA79^+^ vessels by immunohistochemistry in a subset of human primary and naïve melanoma and breast, ovarian, colon, and lung tumor sections. They further confirmed with additional human HEV-specific marker HECA-452 (72) and human HEV-specific antibodies G72 and G152 (73) that these vessels phenotypically resembled LN-HEVs and thus, termed them tumor HEVs (TU-HEV). Importantly, TU-HEVs were specifically located within lymphocyte-rich areas and frequently contained luminally-attached or extravasating CD3^+^ cells. Indeed, the density of TU-HEVs in breast cancer was a predictor of CD3 T cell and B cell infiltration, suggesting that TU-HEVs, like their homologs in LNs, are major gateways for lymphocyte infiltration (26). Importantly, the density of TU-HEVs positively correlated with disease-free, metastasis-free, and overall survival rates in a retrospective cohort of primary breast cancer patients, thus suggesting their implication in the formation of immune-active TLS-like structure (74).

To date, these seminal results have been confirmed by other groups in breast cancer (75) and extended to a broader panel of other human cancers (76–79). Thus, MECA79^+^ vessel-containing lymphocyte aggregates were described in renal (80), gastric (81), pancreatic (82–84), and head and neck carcinomas (85–88), among many other cancer types (89).

Although these studies defined a common TU-HEV phenotype by MECA79-positivity across the different human tumor types, they also described a more heterogeneous phenotype in comparison to that of LN-HEVs. For instance, in lung cancer, MECA79^+^ blood vessels were also shown to express high levels of MAdCAM-1 (78). Additionally, in human melanoma (90) and oral squamous cell carcinoma (85, 88), the typical thick MECA79^+^ vasculature with cuboidal ECs coexists with thin-walled MECA79^+^ vessels displaying a flattened EC morphology and dilated lumens. It is conceivable that these observations could reflect different degrees and stages of TU-HEV maturation, thus implying functional differences among intratumoral MECA79^+^ vessels. Indeed, plump TU-HEVs, that are surrounded by substantial lymphocyte aggregates are thought to be more mature than some isolated and flat TU-HEVs located at the periphery.

Since these observations are, however, only correlative, there is still a debate to which extent TU-HEVs are necessary to actively influence cancer progression in TLSs or TLS-like structures. Certainly, there are discrepancies between studies that are not only inherent to the considered tumor type but also dependent on intratumoral heterogeneity of TU-HEVs and TLSs, respectively. For instance, TU-HEVs can be present in T cell- and DC-rich areas (74, 91) while also present in B cell-rich areas (92, 93). Moreover, TU-HEVs appear to be more frequent than TLSs in breast cancer (26, 94) and melanoma (79, 91). Thus, it appears that the presence of TU-HEVs does not always correlate with *bona fide* intratumoral TLSs that inherit a “strict” definition but instead with a broader spectrum of TLS-like structures (23).

As the correlation of spontaneous TU-HEV and TLS formation with a positive outcome is preferentially observed in specific cancer types, one can envision that these naïve cancers have obtained a permissive environment for ectopic HEV formation. In line with this idea, “hot” tumors may be more prone to TU-HEV formation while “cold” tumors remain anergic (95).

This further raises the question as to whether cancer therapies and specifically those generating an immune-stimulating reaction, can instigate HEV and TLS formation. So far, only a few reports in breast (75, 96) and colorectal (97) tumors have correlated the presence of tumoral TLSs/HEVs with a favorable response to combined radio- and chemotherapy (22). Given the plethora of ongoing clinical trials evaluating the effects of immune checkpoint inhibitors (ICI), it is of great interest to evaluate thoroughly TU-HEV/TLS formation and its correlation with patient response. In support, higher TLS density in tumors correlated with an improved response to ICIs and increased survival in melanoma and soft-tissue sarcoma patients (92, 93, 98),

In summary, there is accumulating evidence from these clinical data that the formation of HEV-containing TLSs can be a marker of good prognosis but whether TU-HEV formation is a prerequisite for instigating TLS formation and antitumor response in human cancer remains obscure.

## Lessons Learned From HEVs in Murine Tumors

### Spontaneous TU-HEV Formation

Why do some tumors spontaneously form HEVs while others do not? One clue comes from the observation that spontaneous HEV formation in tumors of mice was only observed when tumor cells expressed strong antigens, i.e., the commonly used OVA-antigen peptide in tumor cell lines or the viral oncoprotein simian virus SV40 large T-antigen to drive endogenous tumor formation in pancreatic islets (99, 100). The presence of such antigens suggests that strongly antigenic tumors may have a more robust lymphocyte activity and, thus, be better poised to instigate TU-HEV formation.

So far, observations of spontaneous TU-HEVs in mice are rare and only reported in B16-OVA melanomas, LLC-OVA lung carcinomas and Rip1Tag5 (RT5) pancreatic neuroendocrine premalignant lesions (99–101). In line with the requirement of a tumor antigen to elicit a robust immune response, expression of SV40 Tag in the beta cells of pancreatic islets in RT5 mice does not commence before 10–12 weeks of age, leading to the recognition of Tag as a nonself protein (102). In contrast, pancreatic beta cells express Tag in Rip1Tag2 (RT2) mice already during embryonic development, probably due to differences in the site of integration of the transgene, and thus become tolerant to Tag (103). As a consequence, Tag expression in RT5 mice causes a severe immune response with intense infiltration of CD4 and CD8 T cells, B cells, and macrophages in hyperplastic RT5 islets, while islets of RT2 mice display a paucity of lymphocytes and do not become inflamed. This leads to the formation of immature MAdCAM-1^+^ HEVs in inflamed RT5 hyperplastic islets but not in non-inflamed RT2 hyperplastic islets suggesting that immune cell infiltrates are required to initiate HEV formation although they appear not to be fully developed (100). Similarly, the spontaneously formed TU-HEVs in B16-OVA melanoma and LLC-OVA exhibited much weaker PNAd positivity compared to LN-HEVs likely reflective of an immature HEV phenotype similar to that observed in RT5 hyperplastic islets (99, 100). What these data also imply is the necessity of reactive immune cells to enable HEV formation in tumors.

### Immune Cells Regulate HEV Neogenesis in Tumors

The first evidence that hematopoietic cells can regulate LN-HEVs in adulthood comes from the study of Moussion and Girard (68). Depleting CD11c^+^ DCs in adult CD11c-DTR mice by administering diphtheria toxin (DTX) degenerated HEVs and reverted them to a MAdCAM-1^+^ immature stage reminiscent of neonatal HEVs. Congruently, CD11c^+^ DCs are crucial for the switch from MAdCAM-1 to MECA79/PNAd expression during neonatal development of peripheral LNs (104). Consequently, due to the reduced HEV ability to recruit lymphocytes into the LN, LN size and cellularity was reduced (68).

**Figure 2:**
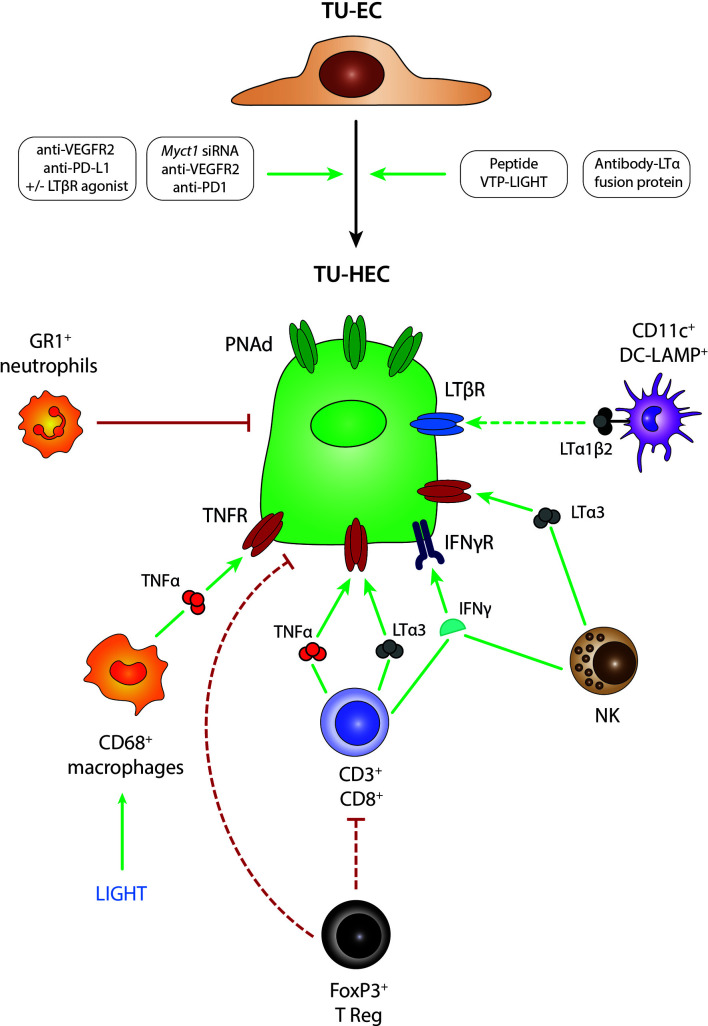
Mechanisms of TU-HEV formation and maintenance. Several treatment regimens in mouse model studies have demonstrated the transition from TU-ECs to TU-HECs expressing PNAd; including 1- the combination of anti-VEGFR2 anti-angiogenic therapy with anti-PD-L1 immunotherapy, 2- the single activation of the LTβR axis with LTβR agonists, 3- the triple combination of anti-VEGFR2 anti-angiogenic therapy with anti-PD-L1 immunotherapy and LTβR agonists, 4- the triple combination of anti-VEGFR2 anti-angiogenic therapy with anti-PD1 immunotherapy and *Myct1* siRNA-mediated silencing, 5- engineered EC-specific VTP peptides expressing LIGHT and 6- fusion protein antibodies delivering LTα to the tumors (antibody-LTα fusion protein). TU-HECs are regulated by specific immune cell types and signaling cues within the tumor microenvironment; including 1- CD3^+^/CD8^+^ T cells *via* TNFα, LTα3 and IFNγ, 2- FoxP3^+^ Tregs that either directly regulate PNAd expression or indirectly by exerting an immune-suppressive effect on CD3^+^CD8^+^ T cells, 3- NK cells *via* LTα3 and IFNγ, 4- CD11c^+^ DC-LAMP^+^ DCs *via* membrane-bound LTα1β2, 4- LIGHT-induced CD68^+^ macrophages *via* TNFα and 5- GR1^+^ neutrophils. TU-EC, tumoral endothelial cell; TU-HEC, tumoral high endothelial cell; PNAd, peripheral node addressin; DC, dendritic cell; NK, Natural Killer cell.

Observations of DC-LAMP^+^ mature DCs in close proximity of TU-HEVs in human breast cancer and melanoma tissue led to the initial proposition that DCs may also regulate HEVs in cancer (74, 105, 106) **(** **Figure 2** **)**. Nevertheless, most of the studies in mouse tumor models point to a more predominant role of lymphocytes. Spontaneous HEVs did not occur in B16-OVA tumors grown in Rag2-/- mice, lacking B and T lymphocytes but appeared when Rag2-/- mice were reconstituted with CD8 T cells before tumor implantation (99). Similarly, CD3 and CD8 T cell depletion led to a reduction of TU-HEV frequency and lymphocyte infiltrates in the pancreatic RIP1-Tag5 and a methylcholanthrene-induced fibrosarcoma tumor models (107, 108). The role of CD8 T cells as critical inducers of TU-HEV formation is further underscored by the observation that depletion of immunosuppressive CD4 T regulatory (Treg) cells renders tumors permissive to TU-HEV and TU-TLS neogenesis (108–110) **(** **Figure 2** **)**. Noteworthy, FoxP3^+^Treg cell depletion with DTX using the FoxP3-DTR system, also disrupted the physiological LN-HEV network (108). DCs were, however, not required to form HEVs in Treg-depleted fibrosarcomas because HEVs were unaffected upon DC depletion (108). Although CD11c is a marker traditionally associated with pan-DCs, the expression of CD11c often overlaps in macrophages and DCs in non-lymphoid tissues (111). Therefore, the depletion of CD11c^+^ cells in the before-mentioned study may not be restricted to the intratumoral DCs. So far, it remains unknown whether Tregs may directly suppress HEV neogenesis by interacting with tumor endothelial cells or indirectly by inhibiting CD4 and CD8 lymphocytes and creating an immunosuppressive environment.

Although lymphocytes appear to be the main regulators of TU-HEV neogenesis, innate immune cells have also been proposed as potential candidates (107, 112). Particularly, CD68^+^ macrophages have also been shown to facilitate TU-HEV formation in the Rip1Tag5 tumor model by producing the TNF receptor ligands TNFα and LTα (107). Moreover, in a Kras (G12D)-driven mouse model of lung cancer, the depletion of GR1^+^ neutrophils increased the intensity of MECA79 staining in CD31^+^ ECs, indicating that Gr1^+^ neutrophils are negative regulators of TU-HEVs (112) **(** **Figure 2** **).**

What are then the signaling pathways in ECs that instigate HEV formation in tumors? So far, it appears that the signaling cues and mechanisms involved in LN-HEV formation are also involved in tumoral HEV neogenesis. Several studies point to the lymphotoxin (LIGHT, LTα1β2)/LTβR pathway as the prevailing signaling cue in inducing TU-HEVs. Treatment with the LTβR agonist or the LTβR ligand LIGHT, which had been targeted to the tumor vasculature by fusing it to a vascular zip code peptide, induced MECA79^+^ HEVs in various mouse tumor models, including those of breast cancer, neuroendocrine pancreatic tumors, and glioblastomas (107, 113–115).

Important to note is that anti-angiogenic immunotherapy in the form of anti-VEGF plus anti-PDL1 induced the noncanonical LTβR pathway in ECs of breast and pancreatic endocrine tumors, which enabled HEV formation, enhanced lymphocyte infiltration, and prolonged survival of tumor-bearing mice (113). The addition of agonistic LTβR antibodies to anti-VEGF plus anti-PDL1 therapy, thus fully activating the LTβR signaling cues, further increased HEV numbers and maturation in breast and pancreatic cancer and sensitized glioblastoma to the therapy. Combination treatment with LTβR antagonists, however, reversed these effects (113, 114) **(** **Figure 2** **)**.

Further, TNFR1 stimulation *via* TNFα or LTα3 seems to be accountable for spontaneous TU-HEV formation independent of LTβR. While LTβR-Ig blockade did not alter spontaneous HEVs in B16-OVA melanomas, HEVs were absent in these tumors when grown in TNFR1/2^-/-^ mice or Rag2^-/-^ mice replenished with LTα^-/-^ CD8 T cells (99). In a carcinogen-induced fibrosarcoma model, Treg depletion increased numbers, proliferation, and activation of TNFα-producing intratumoral CD8^+^ T cells, which then induced the formation of intratumoral HEVs in a TNFR-dependent manner. Blockade of TNFR with TNFRII.Ig, anti-TNF antibodies, or *via* anti-LTα treatment reduced TU-HEV areas specifically in Treg-depleted fibrosarcomas, while LTbR-Ig had no effect (108). Targeting an LTα fusion protein to the tumor site has been shown to be another strategy to successfully induce MECA79^+^ HEVs and lymphoid aggregates in the tumor microenvironment. In this study, electron microscopy observations confirmed the HEV morphology of around 30% of the blood vessels. Moreover, the therapy was efficient in eradicating subcutaneous B78-D14 melanomas and their established pulmonary metastases (116). These observations are in line with a study conducted in chronic inflammation where the transgenic expression of LTα under the control of a rat insulin promoter generated structures resembling lymph nodes concerning the cellular composition and HEV detection (117).

Another potential signaling molecule involved in HEV formation is IFNγ produced by NK cells and T cells because it stimulates the expression of the CXCR3 ligands CXCL9 and CXCL10, and the CCR7 ligand CCL21 as well as ICAM-1 in ECs, which all together induce T cell recruitment and infiltration (118). Although IFNγ is not sufficient to directly induce HEV neogenesis (99), it may have supporting functions in instigating TU-HEVs by increasing lymphocyte influx. This may have important implications because the signaling pathways described above, induce vessel normalization. During this process, excessive immature tumor vessels become pruned, lymphocyte adhesion molecules increase, and pericytes align more closely to and stabilize the vasculature which leads to enhanced blood flow and T-cell infiltration. Vessel-targeted LIGHT normalized blood vessels in murine primary tumors and metastases (107, 114, 115, 119) and antiangiogenic therapy, alone and in combination with checkpoint blockade induced vessel normalization and boosted by further activation of the LTβR signaling using a LTβR agonistic antibody (113). In addition, a recent study has shown that genetic deletion of Myct1, a direct target gene of ETV2, was sufficient to normalize tumor vessels and induce TU-HEV formation in subcutaneous sarcoma, concomitant with antitumoral immunity. Myct1 deletion combined with immunotherapy was successful in increasing long-term survival in anti-PD1 refractory breast cancer model (120).

Thus, although it remains obscure whether vessel normalization is a prerequisite for HEV formation, it is tempting to speculate that vessel normalization in tumors is a trigger to enhance lymphocyte infiltration which in certain areas reaches a signaling threshold that could lead to HEV neogenesis.

What these studies also reveal is that the complex process of TU-HEV development likely involves multiple pathways and signals, and requires further investigation. It is plausible that a process similar to the proposed two-step differentiation model of HEV formation in chronic inflammation, may take place. In accordance with this model, TNFR1 is required in the initial stages of chronic inflammation and induces flat MECA79^+^ blood vessels, whereas the LTβR pathway is involved for the additional maturation and acquisition of a fully mature HEV phenotype (121, 122).

### Do Tumoral HEVs Generate Specific Immune-Reactive Centers?

Naïve T cells are thought to become primed and activated by tumor antigen-presenting DCs, expand and differentiate in the tumor-draining lymph node, also referred to as sentinel LN, from which they home to the tumor site (123).

Interestingly, analysis of T cell clonality and homing indicate that TU-HEVs can facilitate infiltration of naïve T cells *via* the selectin L/CD62L axis into the tumor (99, 116). T cell activation, therefore, not only occurs in the sentinel LN, but may also take place at the tumor site (22, 116, 124). The recruitment of naïve T cells into the tumor, bypassing the activation in the sentinel LN, may help to speed up and favor the generation of an *in situ* antitumoral response but also requires antigen presentation by DCs and other APCs for T cell activation (125). Congruently, TLSs have been shown to facilitate interactions between T cells and tumor-antigen-presenting CD11c^+^ DCs in a genetically engineered mouse model of lung adenocarcinoma. Staining of γ-tubulin (a marker of the microtubule-organizing center [MTOC]) depicted immunological synapses between DCs and CD8 T cells, in the tumors, which in turn upregulated the early activation marker (CD69) and became proliferative (109). The concept that naïve T cells may be educated within the tumor has also been observed in human tumors. Mature LAMP^+^ DCs closely associated with CD3 T-cells have been identified in juxtaposition to TU-HEVs in human breast cancer (74). Importantly, dense aggregates of MHC-II^+^ APCs and CD8 T cells have been identified in human renal cell carcinomas (RCC). These niches contain TCF1^+^PD1^+^stem-like CD8 T-cells that undergo slow self-renewal and give rise to terminally differentiated CD8 T cells. They provide the proliferative burst and thereby foster the antitumoral immune response seen after anti-PD1-immunotherapy (126, 127). Interestingly, these T cell-enriched nests appear to be active immune centers that closely resembled the extrafollicular regions of the lymph node and were quite distinct from the typical B cell-enriched-identified TLSs found in RCCs which did not exhibit closely interacting DCs and T cells (126). Whether TU-HEVs are also an integral part of these APC niches remains to be investigated.

Besides therapeutically exploiting TU-HEVs as lymphocyte gateways, they also offer a “route” to deliver chemotherapeutic agents. One of the key features of the pancreatic ductal adenocarcinoma (PDAC) is the dense and poor vascularized microenvironment which limits the penetrance of drugs to the site of the tumor. TU-HEVs have been identified in the stroma of human PDAC implanted in a humanized mouse model (84). Targeting TU-HEVs with MECA79-Taxol-nanoparticles has been shown to improve efficacy in delivering Paclitaxel to the tumor, resulting in tumor growth inhibition (84). Similarly, in preclinical models of breast as well as pancreatic tumors, an antibody (MHA112)-based strategy has been used to directly deliver the chemotherapeutic agent to tumors *via* targeting of TU-HEVs (128). Given these results, combining HEV-inducing strategies with HEV-specific deliverables of chemotherapeutical agents may represent a synergistic approach for future cancer therapy.

## HEV Alterations in Sentinel LNS

LNs are critical for immune surveillance, providing a highly organized hub to obtain optimal conditions for naïve lymphocytes to interact with APCs. In response to certain stimuli such as infection and inflammation, the draining LNs undergo considerable expansion, known as lymphadenopathy, to accommodate the increased need of lymphocyte priming. This process is characterized by increased blood flow and lymphocyte trafficking while the lymphocyte exit *via* lymphatics is temporarily blocked (129–131). These changes increase the probability of antigen presentation and ensure the initiation of the appropriate antigen-specific immunity. LN expansion is orchestrated by transient LN-vasculature remodeling. Upon inflammation, HEVs quickly expand by undergoing clonal proliferation of a putative progenitor cell and succumb upon cessation of inflammation to return to their homeostatic stage (132). LN-HEV plasticity and remodeling upon inflammation are controlled by extensive reprogramming and have been comprehensively investigated at the transcriptional level (59).

**Figure 3:**
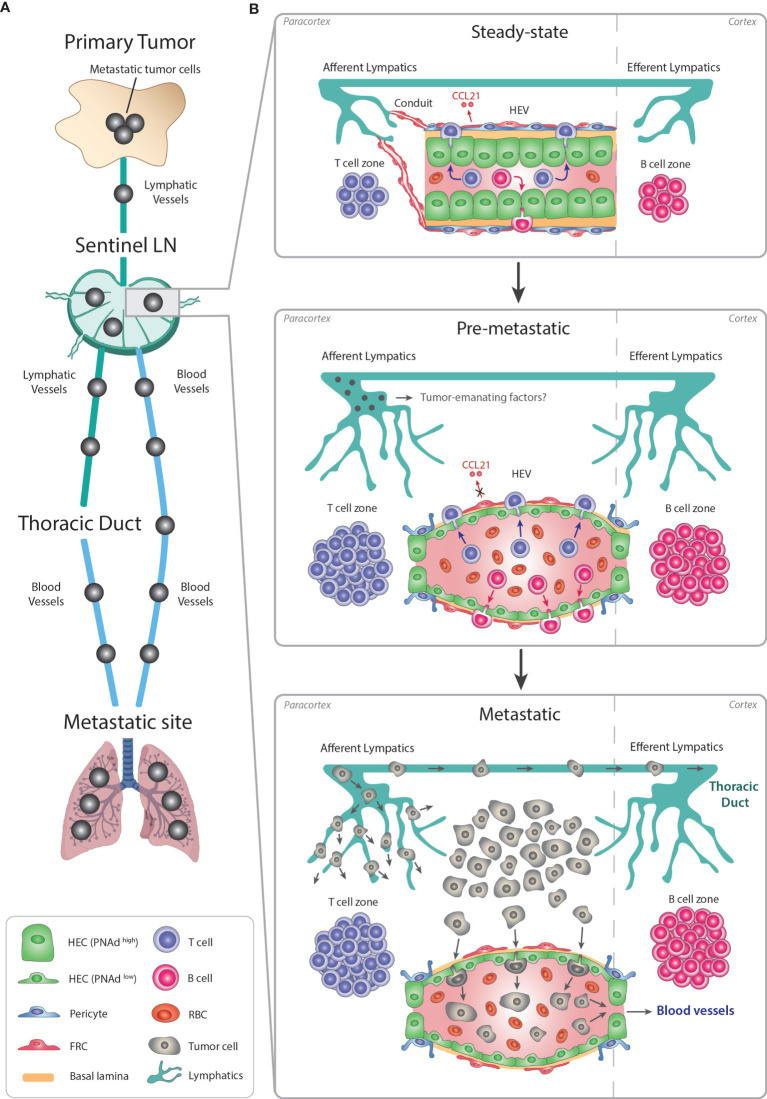
Remodeling of LN-HEVs during cancer metastasis. **(A)** Metastasis is a stepwise process leading to the dissemination of cancer cells from the primary tumor towards preferential metastatic sites. Most commonly, metastatic cancer cells use the lymphatic system to exit the primary tumor, reach a proximal sentinel LN, circulate into adjacent LNs and eventually drain from the thoracic duct into the systemic venous system, thus spreading towards metastatic sites (e.g., lungs). Metastatic tumor cells in the LN parenchyma can also directly intravasate into the bloodstream *via* LN-HEVs and disseminate towards metastatic sites. **(B)** This alternative route involves an important remodeling of the sentinel LN already at the pre-metastatic stage, in preparation for the arrival of cancer cells. Overall, the sentinel LN expands, as evidenced by 1- expanded T and B cell compartments in the paracortex and cortex, respectively, and by 2- an extensive lymphangiogenesis. Importantly, HEVs are remodeled as 1- HECs switch to a PNAd^low^ flat phenotype with thin-walled basal lamina and enlarged lumens filled with numerous RBCs, and 2- CCL21 expression is dysregulated in FRCs, suggesting impaired lymphocyte-recruiting functions of sentinel LN-HEVs. Whether tumor cells from the primary site secrete specific factors preparing this pre-metastatic niche prior to their circulation into the sentinel LN remains to be elucidated. Altogether, the expanded sentinel LN and remodeled HEVs allow the direct spreading of cancer cells from afferent lymphatics into the venous bloodstream during the metastatic stage. LN, lymph node; HEV, high endothelial venule; HEC, high endothelial cell; RBC, red blood cell; FRC, fibroblastic reticular cell.

Sentinel LNs are considered the major site at which the anti-tumoral immunity is initiated, but they also represent a privileged site for cancer cell dissemination (133) **(** **Figure 3** **)**.

Similar to inflamed LNs, sentinel LNs also undergo vascular remodeling (88, 134–137). Sentinel LN-HEVs often show dramatic morphological changes, shifting from thick-walled blood vessels with a small lumen to a thin-walled vasculature with an enlarged lumen and abundant red blood cells (RBC). Moreover, HEVs of sentinel LNs can display loss in PNAd/MECA79 expression in association with dysregulation of CCL21 in perivascular FRCs (134–136). Given the importance of PNAd and CCL21 in the recruitment remodeling of naïve T cells and initiation of the adaptive response, the dysregulation of these components in sentinel LNs may indicate impaired LN functionality.

Noteworthy, experiments in nude mice have shown that these dramatic changes occur only in tumor-reactive LNs and not in endotoxin-induced lymphadenopathy, indicating that the mechanism of vascular reorganization in sentinel LN may differ from that of inflammatory-reactive LNs. Importantly, these studies have also shown that T cells are not the major players in the vascular remodeling of sentinel LN (135).

HEV abnormality has been observed in sentinel LNs of breast cancer, melanoma, and squamous cell cancer patients (88, 134–137). As these modifications occur before detection of metastatic cancer cells in the sentinel LN (135, 136), it is conceivable that tumor-emanating factors induce LN-HEV alterations to establish a pre-metastatic niche permissive for tumor cells. One could also speculate that the presence of enlarged HEV lumen engorged with RBC, could enhance oxygen and nutrient delivery for arriving cancer cells.

The majority of cancers invade the sentinel LN *via* lymphatic vessels before spreading to distant organs (138). Until recently, it was expected that metastatic cancer cells would also leave the LN through the efferent lymphatic vessels, the LNs of higher echelons, and the thoracic duct (139) **(** **Figure 3** **)**. However, two recent seminal studies in mice have revealed that cancer cell dissemination can occur through the LN-HEVs by intravital microscopy. In the first study, murine 4T1 breast cancer cells intra-lymphatically infused into the subcapsular sinus of pLNs, migrated towards the LN center, then localized around HEVs, transmigrated through HEVs, and subsequently disseminated into the lungs. Importantly, lymphatic ligation did not compromise the capability of cancer cells to colonize the distal organs (140). Similar results were obtained in the second study, in which, using time-lapse multiphoton intravital microscopy, the photo-converted metastatic cancer cells were first seen in the subcapsular sinus and later invaded the cortex of the LN where they transmigrated into HEV^+^ vessels. Metastatic cancer cells were then eventually detected in the systemic blood circulation and in the lungs (141).

Overall, these experimental studies revealed that LN-HEVs serve as a gateway not only for lymphocyte trafficking into the LN but can also enable tumor cell intravasation into the bloodstream. Concomitantly, HEV alterations into flattened, dilated blood vessels occur that have lost their morphological and likely functional properties and may likely be induced by the tumor. To this end, the implication of tumor-emanating factors in HEV remodeling in the premetastatic niche in LNs is unknown (**Figure 3**). In addition, whether tumor cell dissemination in human LNs also occurs through HEVs, remains to be clarified, but substantial LN-HEV remodeling preceding LN metastasis has also been shown in human breast cancer patients (136). The premetastatic LN alterations also provide an opportunity for identifying biomarkers of vascular changes in sentinel LNs that could be used to predict disease progression in human cancer (136).

## Concluding Remarks and Future Directions

Since sufficient infiltration of intratumoral T cell effector cells in malignant lesions is a major hurdle in anti-cancer immunotherapy (11, 142), therapeutic induction of HEVs represents a compelling approach to boost effective transmigration of lymphocytes into the tumor. This may increase the benefits of immune checkpoint blockade and improve cell-based immunotherapies using chimeric antigen receptor (CAR) T cells in solid tumors. An additional and specific advantage of therapeutic HEV induction may be the creation of immune-reactive niches that spurt T cell activation and differentiation and replace exhausted and dysfunctional effector T cells.

Although these are tantalizing concepts, they also raise several questions about the tumor-specific ontogeny, regulation, and function of HEVs. Studies in mouse tumor models have provided the first insight into the cellular and molecular regulators of HEV formation and maintenance, partly resembling those of LN-HEVs and partly depicting disparities. The varying degrees of HEV morphology in tumors may also affect HEV functionality, as shown in sentinel LNs, raising concerns about the implication of HEVs in recruiting tolerance-promoting lymphocytes in tumors. Indeed, TLSs are correlated with a worse prognosis in some tumor types, including hepatocellular carcinomas, RCC lung metastases, and head and neck cancer, although the reasons are unknown (22, 143, 144).

An accumulation of Tregs has been observed in TLSs of a lung cancer mouse model (109). However, Treg depletion enhanced HEVs and improved an immune response in these tumors (109), as also observed in fibrosarcoma (108). Recent single-cell transcriptomic analyses of homeostatic and inflamed LNs (59, 145) have provided a specific transcriptional signature of LN-HEVs that has shed some light on LN-HEV-specific signals (146). Comparing transcriptomics between LN-HEVs and TU-HEVs will be important to inform about general and tumor-specific HEV characteristics and functions. To this end, HEV development in LNs and in tumors remains obscure. When LNs become inflamed and enlarged, HEVs quickly expand in part by progenitor cell propagation but by what means HEVs arise from tumor endothelial cells and expand is unknown. Such knowledge, however, will be crucial to therapeutically switch on and off HEV formation in a controlled manner in malignant lesions to avoid potential autoimmune reactions.

Finally, *in situ* HEV model systems can help to dissect the cellular and molecular circuits controlling TU-HEV neogenesis. To date, nonetheless, HEVs cannot be cultured and maintained *ex vivo*, thus rendering mechanistic analyses difficult. Indeed, several attempts to culture purified HEV-ECs have failed due to a rapid loss of their unique features once plated as monolayers suggesting the necessity of additional cell types, factors and specific growth conditions (147–150). One attractive model system may be *bona fide* vascular organoids that have been successfully generated from human ES cells and fully recapitulate the heterogeneity and functionality of vessels *in vitro* and *in vivo* upon transplantation (151–153). Other systems involve microfluidics (154) or EC reprogramming (155) which could serve as more relevant platforms to induce and maintain HEV *ex vivo*.

High endothelial venules display a unique specialization of blood endothelial cells and due to their explicit interaction with lymphocytes, only arise in specific lymphoid organs during development. The fact that they can also ectopically develop in non-lymphoid organs during chronic (tumor) inflammation in the adult is again linked to their intimate relationship with lymphocytes, which may go far beyond mere lymphocyte transportation. Looking into the future, further investigations of TU-HEV blood vessels are timely to better comprehend their nature and functionality because enabling their therapeutic induction in tumors offers promising avenues, not only for immunotherapies, but also for other types of cancer treatment.

## Author Contributions

All authors contributed to the article and approved the submitted version.

## Funding

This work was supported by grants from the Flemish government FWO (G0A0818N to GV and GB, G072021N to SG and GB) and the National Institute of Health NIH/NCI (R01CA201537 to GB).

## Conflict of Interest

The authors declare that the research was conducted in the absence of any commercial or financial relationships that could be construed as a potential conflict of interest.

## Publisher’s Note

All claims expressed in this article are solely those of the authors and do not necessarily represent those of their affiliated organizations, or those of the publisher, the editors and the reviewers. Any product that may be evaluated in this article, or claim that may be made by its manufacturer, is not guaranteed or endorsed by the publisher.
